# The adequacy of antenatal care services among slum residents in Addis Ababa, Ethiopia

**DOI:** 10.1186/s12884-016-0930-z

**Published:** 2016-06-15

**Authors:** Yibeltal T. Bayou, Yohana S. Mashalla, Gloria Thupayagale-Tshweneagae

**Affiliations:** Department of Health Studies, University of South Africa, Pretoria, South Africa

**Keywords:** Addis Ababa, Adequacy of antenatal care, Slum residents

## Abstract

**Background:**

There are recent efforts made to eliminate inequalities in the utilisation of basic health care services. More emphasis is given for improvement of health in developing countries including maternal and child health. However, disparities for the fast-growing population of urban poor are masked by the urban averages. The aim of this paper is to report on the findings of antenatal care adequacy among slum residents in Addis Ababa, Ethiopia.

**Methods:**

This was a quantitative and cross-sectional community based study design which employed a stratified two-stage cluster sampling technique to determine the sample. Data was collected using structured questionnaire administered to 870 women aged 15–49 years. Weighted ‘backward selection’ logistic regression models were employed to identify predictors of adequacy of antenatal care.

**Results:**

Majority of slum residents did not have adequate antenatal care services with only 50.3, 20.2 and 11.0 % of the slum resident women initiated antenatal care early, received adequate antenatal care service contents and had overall adequate antenatal care services respectively. Educational status and place of ANC visits were important determinant factors for adequacy of ANC in the study area. Women with secondary and above educational status were 2.7 times more likely to receive overall adequate care compared to those with no formal education. Similarly, clients of private healthcare facilities were 2.2 times respectively more likely to receive overall adequate antenatal care compared to those clients of public healthcare facilities.

**Conclusion:**

In order to improve ANC adequacy in the study area, the policy-making, planning, and implementation processes should address the poor adequacy of ANC among the disadvantaged groups in particular and the slum residents in general.

## Background

Antenatal care (ANC) programmes were introduced at around the beginning of the 21st century as a constellation of interventions that a pregnant woman receives from health care services [1]. ANC aims to prevent and identify pregnancy risks and treat conditions timely through providing appropriate information to the client [2, 3]; and helps a woman to approach pregnancy and birth as positive experiences. Hence, the care should be appropriate, affordable and fulfil the needs of the mother including the poor [3–5]. As cited in Garrido [6], the Berg’s report on Prenatal Care in Developing Countries indicates the four components of the ANC goal: (1) early detection of pregnant women at risk of any potential complications; (2) action in order to prevent any future difficulties; (3) diagnosis and treatment of pre-existing medical conditions and (4) prompt referral to the appropriate specialist when complications develop during pregnancy.

Comprehensive skilled antenatal care services can save lives of mothers and neonates [3, 5, 7]. There are recommended ANC packages that should be provided to pregnant women regardless of the gestational age at first visit. The packages include physical examinations (weight, height, blood pressure measurement, foetal heartbeat assessment), laboratory investigations (urine and blood samples), preventive procedures (tetanus injection and iron supplementation) and provision of information or counselling on signs of pregnancy complications and measures to be taken by the mother [4, 8].

There are recent efforts made to eliminate inequalities in the utilisation of basic health care services. More emphasis is given for improvement of health in developing countries including maternal and child health. However, there are still huge disparities in maternal health care between different geographic areas and social groups [9, 10].

The number and timing of antenatal visits and the content of services during antenatal visits matter most in identifying pregnancy risks and management of delivery complications [11]. According to WHO, under normal circumstances a woman should have at least four antenatal care visits and the first visit should take place at or before the first 12 weeks of gestation. However, in 2011 only half of pregnant women in the developing regions received the recommended minimum of four antenatal care visits. In the Sub Saharan Africa, the proportion of women who attended four or more antenatal visits has actually fallen from 50 % in 1990 to 45 % in 2010 [5]. Kinney and his colleagues [12] state that low coverage, poor quality and inequities in the provision of essential maternal health care services are existing challenges for Sub Saharan Africa. Remote communities often have poorer access to health care than urban dwellers, and even more privileged are those living in large cities or in the capital [7]. However, the disparities for the fast-growing population of urban poor who struggle as much as or even more than their rural counterparts to access quality health care tend to be masked by the urban averages [12]. Observing all-urban averages based on large data sets such as DHS mask the real picture of inequities in health status and access to healthcare services between the poor and non-poor groups. Hence, programmers would believe that urban population is more advantaged compared to the rural average unless such data sets are disaggregated by socio-economic status [13].

There are well documented socio-economic and demographic factors that affect utilisation of maternal healthcare services. Maternal age is often indicated as a proxy for health care seeking behaviour. Evidences show that older women are more likely to use health care services [11] due to their accumulated experience in using health services, confidence in household decision-making, and might have been told by health worker that older age is a risk factor [7]. However, it is also true that in some societies maternal health care utilisation is lower among older women [9, 14]. There is a thought that older women may consist of more traditional cohorts and may be resisting modern health care services [7].

Unintended pregnancies have been acknowledged as important factors influencing maternal health care seeking behaviour. Studies show that unintended births are associated with delayed initiation of antenatal care [15, 16]; smoking during pregnancy associated with low birth weight, not breastfeeding the baby, and poorer outcomes for the mother and the mother-child relationship. A meta-analysis based on studies conducted in different countries revealed higher odds of inadequate ANC utilisation among women with unintended pregnancies [15].

Education is one of the key social determinants of health and health care. Low levels of education [17] and lack of empowerment prevent women from seeking maternal care [12]. Large disparities in maternal health care exist between rich and poor people [18, 19], between public and private health sectors, between provinces and districts, and among rural, urban, and peri-urban populations [2, 19].

Empirical findings from studies of preventive and curative services indicate that health care utilisation is related to the availability and cost of care during pregnancy and childbirth [20]. Weak health system infrastructures, lack of skilled service providers and substandard quality of care hinder maternal health care utilisation in developing countries [18, 19]. Providing access to health services according to need has become more complex in the context of an increasing role for private providers and, frequently, a more limited role for the public sector [20]. Even in areas with a wide range of public and private health care providers, mothers have limited alternatives to seek care if health systems are characterized by high out-of-pocket payments [12]. Some studies have shown that although women report better quality of care in private facilities, the costs deter them from using the services [7]. For example, a study in Nigeria indicates that low service cost is associated with public hospitals for antenatal care [21]. Despite this, a preference for the use of private providers is still evident even among those guaranteed free access to public providers [20].

Previous studies conducted on ANC and delivery care utilisation in Addis Ababa, Ethiopia have either been facility based, used only one or two single outcome variables (timing and number of visits), focused on the aggregate urban population, or generally scarce [10, 22]. There is no evidence of any study that addressed the overall adequacy of ANC i.e. the content of services, timing of first visit and number of visits in the capital which signals that the factors that affect women’s maternal health care seeking behaviour in relation to adequacy of antenatal care services, mode of delivery and their preference to places of delivery have not been fully studied. This study aims to systematically explore the differences in the maternal health care utilisation among the different community groups and the factors that influence women’s health care seeking behaviour and selection of place of delivery. It is envisaged that a clear understanding of such factors is key in building a responsive maternal health care system and to improve health outcomes in Ethiopia.

## Methods

### Study population

The target population for this study was all women aged 15–49 years living in Addis Ababa, the capital city of Ethiopia. To be included in the study, women should have experienced at least one birth in the last 1–3 years before the date of data collection.

### Sampling

The study employed a stratified, two-stage cluster design. In the urban Addis Ababa, stratification was achieved by using the sub-Cities (10 strata). In the first stage the sampling frame was the lists of clusters (enumeration areas) per stratum. Then using the 2007 Population and Housing Census data, 30 sample points known as enumeration areas (EAs) were selected independently from all the strata with Probability Proportional to Size (PPS) of households in each stratum. PPS is a special and efficient method in multistage cluster sampling [23]. Random sample selection was done using the number of households identified per EA during the 2011 EDHS [9]. The sampling frame in the second stage was the lists of households in each EA. Households (HHs) were the sampling units from which 906 households were selected using systematic random sampling technique i.e., approximately equal numbers of households (30 HHs) in each EA. The final valid sample size was 903 because of non-response. A 10 % adjustment was made during sample size calculation for potential non-response and reporting errors.

### Data collection

Data was collected from December 2013 to January 2014. A questionnaire was developed by the researchers to assess adequacy of antenatal care among slum residents in Addis Ababa, Ethiopia. The questionnaire asked women about their most recent births and a list of questions were also asked to elicit the adequacy of antenatal care among study participants. Demographic and socioeconomic information was also included in the questionnaire. Prior to administration of the questionnaire, it was pilot tested with 15 women that have similar characteristics with the study population but among those outside the selected enumeration areas who were not included in the final sample. The questionnaire was also translated into Amharic the local language mainly used in Addis Ababa.

### Description of independent variables

This study was grounded on the basis of Andersen’s Health care Utilisation Model also known as the Behavioural Model of Health Services [24]. The Andersen Health care Utilisation Model has been used extensively in analysing factors that influence utilisation of health care services and to understand disparities in utilisation of medical services [25]. The model was initially developed to assist in understanding why families use health services, to define and measure equitable access to health care, and to assist in developing policies to promote equitable access [24, 26]. In the analysis of social and individual determinants of health services use among families, the model presupposes that health care utilisation is a function of multilevel factors called *predisposing, enabling or need factors* i.e. individual’s predisposition and the ability or need to use services. The latest framework shows the link between societal determinants, health care system and individual determinants and their impact on health care utilisation [26].

The independent variables for this study were selected based on the modified version of the Behavioural Model of Health Services [26]. The predisposing factors including age, number of living children, current marital status and pregnancy intention related to last childbirth, social structure variables such as education, occupation and ethnicity were considered at individual and household levels. As regards to pregnancy intention, women were asked about their recent birth whether they wanted it then, wanted later, or did not want to have any more children at all. In the analyses, pregnancy intention for the last birth was further defined as a dichotomy variable: *intended* for births wanted by then versus *unintended* for either mistimed or unwanted by then. Women’s education was defined here as the highest level of schooling attended regardless of whether the woman completed the level. Educational status was categorized as no education, primary education and secondary or higher education. Mother’s occupational status was categorized as employed and unemployed.

In 2005, the government of Ethiopia stipulated a package of free maternity and selected child health services [27]. However; women continue to be charged for card fees, medical consultations, specific procedures, and supplies or medications. According to the Safe Motherhood Community-Based Survey, service fees are known to be major obstacles for women and their families when they need medical attention [28]. It is not also uncommon for women to be referred for laboratory investigations to private facilities as the public health centres and hospitals do not have all the necessary services that are included in the free maternity services. Therefore, as enabling factors, individual and family resource indicator variables including health insurance and wealth quintile were included in this study. Those who visited health facility for ANC were asked whether there was an organization or agency that either partially or fully covered their expenses and responses were grouped as ‘yes’ or ‘no’. The relative economic status of the households was determined indirectly through the creation of a wealth index. The construction of wealth index was done using principal components analysis (PCA) via a collection of indicators representing durable goods owned by the household, materials used in construction of the home, water and sanitation facilities and size of the home.

A community resource variable, type of resident was also used in the analysis. Type of resident was categorized based on the five indicators developed by the United Nation Human Settlements Programme (UN-Habitat) [29]. Access to improved water, access to improved sanitation, sufficient living area, durability of housing and secure tenure (housing tenure) were used in the construction of type of resident. According to UN-Habitat a household is categorized as *non-slum* if all of the above five indicators are fulfilled, otherwise *slum*. Of the 903 respondents, 870 were slum residents and the remaining 66 were either non-slum households or missing. Hence, this paper focuses on the slum residents only.

A woman meeting at least one of the following criteria was classified as having history of high-risk pregnancy i.e., more than four previous births; history of spontaneous abortion, known high blood pressure, diabetes, or epilepsy was considered high-risk [30].

### Outcome variable, overall adequacy of ANC

In this study, a single overall ANC adequacy indicator was constructed using the three ANC utilization indicators i.e.*, timing of first visit*, *number of visits*, and *adequacy of service content*. Timing of visit was considered adequate if the first visit took place within the first 12 weeks; and the number of visits was considered adequate if the mother had at least four visits in the pregnancy period.

To assess service content, participants were asked about the basic ANC components received as recommended by WHO for all women regardless of the gestational age at first visit to clinics [4]. Information on mother’s weight and height, blood pressure, fundal or uterine height and fetal heartbeat, urine and blood sample taken (blood type, haemoglobin (anaemia) and syphilis test), tetanus injection, iron supplementation, and information or counselling given about signs of pregnancy complications- abdominal pain, severe headache, vaginal bleeding was obtained from respondents. Service content was categorized as *adequate* if all the above services were provided to the mother according to the national recommendation, at least once during the last pregnancy, otherwise *inadequate*. However, it should be noted that women may not specifically know the procedures, examinations or laboratory investigations done for them during antenatal visit.

Although the 1–3 years recall period for this study is shorter than that for Demographic and Health Surveys (up to five years), it still represents a lengthy recall period not far from the recall period of up to two years by the United Nations Children’s Fund supported Multiple Indicator Cluster Surveys (MICS) [9, 31]. Due to inadequate routine health information system usually, national and international monitoring systems have relied on community based survey data like DHS and MICS. Indicators recommended for the DHS and MICS surveys are those that showed accurate reporting at both the individual and population levels. The data collection instrument for this study was developed based on the DHS women’s questionnaire. Population survey validity studies on antenatal and delivery care from China (recall of five years) and Mozambique (recall of 8–10 months) showed accuracy measure ranging from 56 to 88 % [31, 32].

Then, ANC service content was combined with timing of initiation of first antenatal visit and number of antenatal visits in estimating the overall service adequacy. Finally, ANC was defined as *overall adequate* if the woman had her first antenatal visit within the first 12 weeks and had at least four antenatal visits and had received the above 12 basic ANC service contents at least once in the last pregnancy period; otherwise *inadequate*.

### Data analysis

In this study, data was entered using the Census and Survey Processing System (CSPro) software and was analysed for both descriptive and inferential statistics using the Statistical Package for Social Sciences (SPSS) version 16.0. Chi-square test was used for descriptive analyses. Logistic regression modelling was undertaken to examine the net effects of set of explanatory variables over the outcome variables and the odds ratios (OR) were adjusted for all other variables. As the data was collected using complex sampling design (two-stage cluster design), there is unequal probability in the selection of study participants. This may in turn lead to biased parameter estimates. To rectify such problems, in this study, sampling weights and clustering was employed for each outcome variable. As there were multiple potential predictor variables of interest for each of the variables of outcomes, backward selection logistic regression modeling was fitted with probability of removal of a variable set at 0.2. Multi-collinearity and interaction effect checks were also done by measuring Variance Inflation Factors (VIF), labelling of outliers and running cross products. Multicollinearity and interaction effects were not observed among the variables included in the models. The estimates of the crude and adjusted odds ratios were fairly similar and this shows that the variables used for adjustment are not confounding variables.

The outcome variables, number and timing, adequacy of services content and overall adequacy of ANC services, were dichotomized with “1” being adequate and “0” being inadequate. Four different models were fitted to investigate the factors predicting the adequacy of ANC services i.e., for timing, number, service content and overall adequacy. For this study, *p*-value <0.05 was considered as statistically significant at 95 % confidence interval. In this study, data are weighted unless otherwise indicated.

## Results

### Adequacy of ANC services utilisation

There were 870 respondents age ranged between 16 and 46 years and the response rate for this study was 99.7 %. In this study, 81.6 % of the ANC clients had four or more visits during their last pregnancy; but only slightly more than half (50.3 %) had started the first antenatal visit in the first trimester. The median timing was around 13 weeks of gestation. A small proportion (19.6 %) of women received adequate contents of antenatal services. Among the 12 basic service components considered in this study, measurements of maternal height, uterine height, and blood pressure; assessment of urine sample, anaemia and syphilis test and tetanus injection were most likely to be missing in women with inadequate services. Consequently, the proportion of women with overall adequate antenatal care was very low at 11 %. Worth mentioning here is that the timing of first antenatal visit and number of antenatal visits did not show statistically significant association with adequacy of service content in the study area. Only 22.4 % of those who initiated ANC first visit within 12 weeks of gestation and 20.7 % of the women who had four or more antenatal visits received all the basic service components.

### Variations in the adequacy of antenatal care services

Table 1 presents the percentage variations in the adequacy of ANC services by demographic and social-structure variables. Overall, young women and those with history of unintended pregnancy were less likely to initiate ANC early, to have adequate number of visits and to receive sufficient service content as well as overall adequate ANC. Married women and those with fewer numbers of living children were more likely to receive adequate ANC services. Being more educated positively and significantly associated with early ANC attendance, adequate number of antenatal visits, receiving sufficient contents of antenatal care services and ultimately receiving overall adequate antenatal care. Similarly, women who were employed were more likely to receive adequate ANC services. With regards to ethnicity, there were differences in the adequacy of ANC but the associations were not statistically significant (*P* < 0.05).

**Table 1:** Percentage (weighted) distribution of ANC utilisation by demographic and social structure characteristics of women, Addis Ababa, January 2014

Variables	At least four ANC visits	Early ANC visit	Adequate service content	Overall adequate ANC
*Demographic variables*				
Age group				
15–24	80.0**	50.6*	17.9	10.5
25–29	86.8	54.5	21.6	11.7
30–49	87.8	44.9	20.1	8.6
Number of living children				
0–2	86.2	53.4***	21.3	11.5
3–6	82.7	40.9	16.9	6.8
Current marital status				
Currently married	89.8***	53.0*	21.7	11.9
Cohabiting/living together	79.5	45.9	16.8	7.6
Never-married/formerly married	67.7	37.9	15.2	5.2
Pregnancy intention				
Unintended	77.4***	39.2***	14.8	5.3**
Intended	88.3	54.2	22.1	12.1
*Social structure variables*				
Mother’s educational status				
No education	76.8***	36.7***	13.9*	6.6***
Primary education	81.1	43.1	16.3	5.5
Secondary or higher education	91.1	59.6	25.0	15.3
Mother’s occupation				
Unemployed	84.5	50.0	19.3	9.5
Employed	88.0	51.6	22.6	13.3
Ethnicity				
Amhara	86.2	53.6	21.3	13.7
Guragie	87.0	43.3	19.2	7.0
Oromo	81.6	46.8	22.9	8.8
Tigrie	93.2	61.6	20.8	13.2
Others	82.4	49.9	10.8	3.7

Note: (Chi-square *p < 0.05; **p < 0.01; ***p < 0.001)

Table 2 shows the associations between household and community resource variables with adequacy of ANC service. With regards to wealth quintile, the differences in ANC coverage across the categories do not show consistent variation and the associations were not statistically significant. However, women in the middle wealth quintile category were better in early initiation, having more frequent visits and receiving adequate ANC service contents. Health insurance coverage and high-risk pregnancy also significantly and positively associated with use of adequate ANC.

**Table 2:** Percentage distribution of ANC utilisation by healthcare and family and community resource characteristics of women, Addis Ababa, January 2014

Variables	At least four ANC visits	Early ANC visit	Adequate service content	Overall adequate ANC
*Family and community resource variables*				
Wealth index				
Low	87.9*	47.4	22.8	9.1
Middle	87.0	53.2	18.4	12.6
High	82.5	51.9	18.4	10.7
Health insurance				
Yes	94.1	61.2*	33.6*	24.6**
No	85.7	49.7	19.6	9.7
*Health and healthcare variables*				
Type of pregnancy				
High-risk	84.8*	48.9	24.2	12.9
Low-risk	85.5	50.7	19.3	9.7
Type of healthcare facility				
Public	86.3	48.9*	18.6	8.6**
Private	91.7	61.1	29.3	22.5
Time of first visit				
Late	79.7***		17.6	
Early	94.5		22.4	
Total	85.4	50.3	20.2	11.0

Note: (Chi-square *p < 0.05; **p < 0.01; ***p < 0.001)

There were statistically significant differences on the overall ANC service adequacy between private and public health facilities. The proportions of women who had initiated ANC within the first trimester were 61.1 % and 48.9 % for private and public healthcare facilities respectively. About 29.3 % of the private facility attendees received the recommended basic ANC service components at least once, compared to 18.6 % for public facility attendees. The overall adequate use of ANC was nearly as twice among private facility attendees (22.5 %) compared to public facility attendees (8.6 %). But, in general, service adequacy was very low in both cases except the number of antenatal visits which was relatively better (91.7 % for private vs 85.4 % for public).

The mother’s knowledge about danger signs of pregnancy showed an effect on the continuity of antenatal care (table not indicated). Women were asked whether they received counselling services on vaginal bleeding, severe headache, dizziness, sustained vomiting, and swelling, loss of foetal movements, convulsion, severe abdominal pain, or fever. Among all antenatal attendees, 87.1 % of those who were counselled about the danger signs of pregnancy had four or more antenatal visits compared to 83.1 % of those who didn’t receive counselling service.

### Determinants of ANC adequacy

Table 3 presents factors associated with timing of first visit, number of visits, ANC service contents and overall adequacy of ANC service from adjusted binary logistic regressions. The table shows only those variables that were retained in the last elimination models of the respective outcome variables. The variables included in the backward selection logistic regression included: demographic variables (age of respondent, parity, marital status, pregnancy intention); social structure variables (respondent’s educational status, respondent’s occupation, ethnic affiliation); family and community resource variables (wealth index and health insurance); and healthcare variables (type of health facility), The statistical levels of significance of differences between the categories for the particular explanatory variables are indicated by asterisks.

**Table 3:** Factors associated with ANC service adequacy- weighted logistic regression (backward selection)-OR [95 % CI], Addis Ababa January 2014

Variables	Indicators of ANC			
	Adequate number of visits	Early initiation	Adequate service content	Overall adequate ANC
Age *(RC = 15-24]*				
25–29		1.08 [0.74, 1.59]		
30–49		0.69 [0.46, 1.05]		
Wealth quintile *(RC = Low)*				
Medium		1.45 [0.96, 2.21]		
High		1.39 [0.98, 1.97]		
Marital status *(RC = Married)*				
Cohabiting/living together	0.39 [0.23, 0.66]***			
Never-married/formerly married	0.38 [0.20, 0.73]**			
Pregnancy intention *(RC = Unintended)*				
Intended	1.59 [0.97, 2.61]	1.55 [1.08, 2.21]*	1.48 [0.93, 2.37]	1.89 [0.917, 3.88]
Educational status *(RC = No education)*				
Primary education	1.16 [0.62, 2.17]	1.20 [0.73, 1.96]	1.07 [0.55, 2.08]	1.00 [0.34, 2.92]
Secondary and above	2.12 [1.07, 4.14]*	2.190 [1.35, 3.55]**	1.68 [0.89, 3.17]	2.69 [1.29, 5.63]*
Housing tenure *(RC = Rental house)*				
Own house		0.70 (0.47, 1.05]		1.02 [0.56, 1.87]
Place of ANC visit (RC = Public facilities)				
Private facilities			1.59 [0.96, 2.63]	2.16 [1.02, 4.49]*
Health insurance *(RC = Yes)*				
No	0.38 [0.09, 1.67]			0.49 [0.21, 1.12]

Note: OR = odds ratio, * *p* < 0.05; **p < 0.01; ***p < 0.001. ANC = antenatal care, CI = confidence interval, rc = reference category

Educational status was identified as pertinent determinant factor in the three models fitted for timing, number and overall adequacy of ANC services. Women with secondary and above educational status were 2.1, 2.2 and 2.7 times more likely to have adequate number of visits, to initiate ANC early and to have overall adequate ANC care respectively compared to those with no formal education. Similarly, women whose last pregnancies were intended were 1.55 times more likely to initiate ANC early i.e., in the first 12 weeks of pregnancy compared to those with unintended pregnancies. Marital status also showed significant association with number of antenatal visits. Accordingly, never-married/formerly married women and those cohabiting/living together (outside formal marriage) were 61 and 62 % less likely to have four or more antenatal visits compared to their married counterparts.

With respect to the effect of healthcare system, women who had ANC follow ups in private facilities were more likely (OR = 2.16) to have overall adequate ANC services compared to clients of public healthcare facilities. None of the predictor variables showed significant net effect on adequacy of service contents.

## Discussion

### Adequacy of antenatal care services

There is no literature available in Addis Ababa or in Ethiopia on the overall adequacy and related determinants of ANC whose results are based on the combined indicators including the time of first visit, the number of visits, and content of ANC services. A previous study which had applied the Andersons Health Behavioural Model focused only on the impact of timing of ANC booking and it was facility based. The current study gives a more comprehensive picture of ANC adequacy and its relations to selected factors in Addis Ababa by indexing three indicators i.e., adequacy of number of antenatal visits, time of initial visit and recommended contents of services received during the ANC visits.

In order to achieve national goals or meet international standards, ANC services should be adequate and in line with the recommendations of national protocols or WHO guidelines. The number and timing of antenatal visits and the content of services during antenatal visits matter the most in identifying pregnancy risks and management of delivery complications [11].

We have observed that the ANC utilisation rate in terms of having at least one visit was relatively higher than the 2011 EDHS [9]. This might be attributed to the time of the data collection which was about three years later than the EDHS report. The estimates of the timing of first visit, and the content of services, and the overall adequacy of ANC in this study were much lower than in other studies [33]. It was however, difficult to make any comparison of these findings with those from similar studies in the study area or the country because there have not been any study conducted using the three indicators to measure adequacy of ANC particularly among slum residents.

Timing of the first ANC visit is an important indicator of ANC adequacy. It is recommended that women should initiate ANC as early as possible, before or on 12 weeks of gestation [4]. Early initiation of ANC allows clients to have adequate number of visits and sufficient services [34, 35] to recognize risk factors during pregnancy and institute appropriate assessment and treatment. In this study, only slightly more than half (50.3 %) of the pregnant women initiated antenatal visit at or before the 12^th^ weeks of gestation. In another study conducted in Addis Ababa only about 40.2 % of the pregnant women started antenatal visits at or before the 12^th^ weeks of gestation [22]. Similarly, 94.5 and 22.4 % of the women who initiated ANC early had at least four ANC visits (adequate) and received adequate service content respectively compared to 79.7 and 17.6 % respectively of those who started late.

According to the focused ANC approach, for normal pregnancies, WHO recommends only four antenatal visits [4] and Ethiopia has been implementing the Focused ANC for years [8]. In this study about two-third (66.2 %) of the ANC clients had five or more antenatal visits and only 18.3 % of them had four antenatal visits. About one-fifth of the antenatal attendees had history of high-risk pregnancy. The percentage of mothers who received adequate number of ANC visits (85.4 %) in the study area is much higher than the average for urban coverage (45.5 %) in Ethiopia [9] and slightly better than that of the informal settlements in Nairobi (67.7 %) and Ouagadougou (22.0 %) [23]. Therefore, relative to the poor timing and less frequent services received, women in Addis Ababa had more frequent antenatal visits which could be attributed to easy accessibility of the services.

The adequacy of the basic antenatal services received was very low (20.2 %) for slum residents. This finding supports findings in a similar study in Uganda [34]. Considering the higher frequency of visits made by antenatal clients, the finding of low service contents received might signal poor service provision at the health facilities. The women may either also not be aware of the services required [36, 37], or may not know what services to request. Selection of services might also have unaffordable economic consequences [38] i.e., women may not afford the cost associated to extra services if it is based on choice or demand.

### Factors determining the utilisation of ANC services

Several previous studies have indicated multiple barriers that obstruct women’s access to maternal care services [7, 36, 39]. Results of the multivariate logistic regression model in this study indicated that only a few variables were significantly associated with adequacy of ANC utilisation including education, intended pregnancy, and attending private healthcare facilities for antenatal care were positively associated with overall ANC adequacy.

Education has been reported as one of the key social determinants of health and healthcare. Women with low levels of education usually have less knowledge about ANC and poor access to the service [17, 39]. Studies done in urban slums of Dhaka, Bangladesh and Mumbai, India identified educational level of the mother and the husband as the major factor influencing ANC utilisation by the mother [40, 41]. We have observed in this study that less-educated women had significantly lower overall adequate use of ANC i.e., they initiated ANC late; had inadequate number of visits; and received poor ANC service content.

Our analysis has demonstrated that pregnancy intention was the only demographic characteristic identified as a factor for overall adequacy of antenatal care. Women whose last pregnancy was unwanted or mistimed were less likely to have overall adequate ANC. About 26.5 % of the women had unintended last pregnancy. Facility based study in Addis Ababa has shown that women with planned pregnancies were more likely to book ANC early [22] as compared to those with unplanned pregnancies. Other studies also show that unintended pregnancies are associated with delayed initiation of antenatal care [15, 16]. An urban based study in the Democratic Republic of Congo shows that women who had unplanned pregnancies were less likely to attend ANC services compared to those who had planned their pregnancies by themselves or jointly with their partners [42]. Unintended pregnancies and births, both mistimed and unwanted ones, have been shown to have grave consequences to the mother and family and are global social and health burdens including the US [43].

The balance between the private and public health care sectors has now become a hot global agenda. Though more educated and wealthier mothers are more likely to go to private hospitals seeking for maternal health care [14], access, equity, quality, efficiency, responsiveness, adherence to national standards or guidelines, and patient outcomes are major agenda of debate [44]. In the Gambia, women received more sufficient services from private facilities than public facilities and were more satisfied with the services they received [45]. In Addis Ababa, though the private health facilities (34 vs 5 hospitals) outnumber that of the public clinics [46], the majority of antenatal attendees visit public healthcare facilities where it is perceived that the quality of care is relatively poor. Regression analysis results indicate that the type of healthcare facility shows a statistically significant association with the overall adequacy of antenatal care among the clients of the private healthcare facilities who received adequate antenatal care services. The finding is supported by findings in a study in Tanzania [11] and points to a statistically significant and positive effect of women’s attendance at private healthcare facilities on the adequacy of ANC.

Some studies have shown that although women report better quality of care in private facilities, the costs deter them from using the services [7]. In this study, education, pregnancy intention, health insurance and type of place of ANC showed significant association in the bivariate analysis. However, none of the predictor variables showed statistically significant effect on ANC service content in the adjusted model. This might be because service content is more influenced by the service providers’ approach which was beyond the scope of this study. Besides, clients might not be entitled to request the types of specific tests and examinations they want for different reasons including lack of awareness, poor provider-client interaction, fear of extra service charge [22], lack of skill of service provider [47], lack of appropriate equipment or supplies and others [48].

## Conclusion and policy implications

We have reported disparities in antenatal care utilization among slum residents in Addis Ababa, Ethiopia. The predisposing and enabling factors played major roles on the adequacy of ANC care services received by pregnant women residents of slums in Addis Ababa. The level of adequacy of antenatal care services however, are masked mainly by the high coverage in the number of antenatal visits. The findings indicate that standards of antenatal care services in Addis Ababa were not met. For an ANC service to be complete and adequate, the minimum requirements for timing and the number of antenatal visits and service content should be met. In order to improve ANC adequacy in the study area, the policymaking, planning, and implementation processes should focus on the poor adequacy of ANC among slum residents. The ability to use healthcare services among women with lower educational status, with unintended pregnancies, clients of public healthcare facilities and unmarried women should be facilitated.

Specific efforts are needed to target and to raise awareness of the women of lower socio-economic status including those with lower educational status about basic antenatal care services.

The majority of the antenatal attendees (92.6 %) visit public healthcare facilities where the quality of care is relatively poor. Because public facilities are the main providers for the general population and particularly for the disadvantaged groups improving the quality of ANC at these facilities is critical. Regular monitoring mechanisms to make sure recommendations of the World Health Organization or the National Obstetrics Management Protocol are met should be put in place.

The current high level of antenatal care coverage of women making at least four ANC visits, while closely watching for unnecessary more than four antenatal visits as per Focused ANC guideline, should be strengthened because it also increases women’s chances of receiving adequate contents of ANC services and the probability of institutional delivery. However, for improved effectiveness, more attention should be paid to the content of care rather than number of visits as performance indicators. A small proportion of the ANC clients received all the basic ANC service contents at least once. This is alarming in the light of high vulnerability of mothers for pregnancy related complications.

Qualitative research is recommended for in-depth understanding of the problem. This study should be replicated in other areas of the country including rural areas using the combinations of indicators to see the levels and trends of the adequacy of ANC service. This could also involve male partners or husbands than considering only women as subject of further study.

Finally, in interpreting this study's findings, it is advisable to consider some of the limitations of the study. The cross-sectional nature of the data doesn’t allow making causal inferences about the relationship between adequacy of ANC and the risk factors except evidencing the strengths of associations between the predictors and outcome of interest variable. It is important to keep in mind that the analysed data includes information reported by mothers only from last pregnancies or childbirths. Hence, with regards to specific service contents received by the respondents during their last antenatal follow up, women may not specifically know the procedures, examinations or laboratory investigations done to them during antenatal visit, and the recall of the respondents may also not be relied upon. Though it is not an established fact, more educated and wealthier women might also be more likely to recall services received from health facilities than less educated or poorer ones. In our study, more than half and about a third of the private facility attendees for ANC had secondary or higher education and were in the rich wealth category (the top 40 %). This study did not collect data about the views and practices of service providers related to quality of services. The study was also limited to the capital city and findings might not reflect the situation to the rest of the country.
